# Short-Term Outcomes of Laparoscopic Rectal Cancer Surgery at a High-Volume Center in Peshawar, Pakistan

**DOI:** 10.7759/cureus.81133

**Published:** 2025-03-25

**Authors:** Hussain Jan Abbasi, Riaz Ahmad, Haider Abid, Saddam Hussain, Sarmad Saeed Aziz, Muhammad Fahd Shah, Irfan Ul Islam Nasir

**Affiliations:** 1 Surgical Oncology, Shaukat Khanum Memorial Cancer Hospital and Research Centre, Peshawar, PAK; 2 Colorectal Surgery, Shaukat Khanum Memorial Cancer Hospital and Research Centre, Peshawar, PAK; 3 Surgery, Shaukat Khanum Memorial Cancer Hospital and Research Centre, Peshawar, PAK; 4 Surgical Oncology, Shaukat Khanum Memorial Cancer Hospital and Research Centre, Lahore, PAK

**Keywords:** disease-free survival, high-volume center, laparoscopic rectal cancer surgery, postoperative complications, short-term outcomes

## Abstract

Background: Rectal cancer is a serious worldwide health issue, and laparoscopic surgery is becoming a common therapeutic choice because of its advantages, which include less pain after surgery, quicker recovery, and oncological results that are comparable to those of open surgery. Even though laparoscopic rectal cancer surgery has proven successful in developed countries, it is still not well-studied in places with low resources, like Pakistan.

Objective: The primary objective of this study was to evaluate the clinical outcomes and surgical efficacy of laparoscopic rectal cancer surgery in 209 patients, focusing on operative details, histopathological findings, and postoperative complications. The secondary objective was to assess 90-day disease-free survival (DFS), mortality rates, and the need for reexploration or readmission.

Materials and methods: This retrospective observational study was performed at the Surgical Oncology department, Shaukat Khanum Memorial Cancer Hospital, Peshawar, a high-volume center for colorectal surgery. Assessment of short-term surgical results of laparoscopic rectal cancer operation ensued from April 1, 2021, to March 31, 2024. The sample comprised 209 patients with rectal cancer who were treated by laparoscopic rectal cancer surgery. A consecutive sampling technique was used to enroll eligible patients during the study duration.

Results: The mean age of the patients was 43.07 ± 22.1 years. The male-to-female ratio was 1.4:1, with males comprising 131 patients (62.7%) and females, 78 patients (37.3%). The regional distribution included 54 (25.8%) from Afghanistan, 11 (5.2%) from the federally administered tribal area (FATA), 141 (67.5%) from Khyber Pakhtunkhwa (KPK), and three (1.4%) from Punjab. Preoperative assessment classified 191 (91.4%) patients as American Society of Anesthesiologists (ASA)-II, and 18 (8.6%) cases as ASA-III.

Conclusion: Laparoscopic rectal cancer surgery demonstrates favorable short-term outcomes, including minimal morbidity, low conversion rates, and promising oncological results in a high-volume center in Peshawar, Pakistan. These findings support the feasibility of laparoscopic surgery for rectal cancer in resource-constrained settings.

## Introduction

Colorectal cancer has been a major global health concern where rectal cancer alone contributes to almost one-third with an estimated 704,000 new cases per year [1]. Major technological developments, especially the systematic shift from open surgery to minimally invasive surgery, have revolutionized rectal cancer treatment. Laparoscopic rectal cancer surgery was developed late in the 20th century and has only recently become the standard of practice due to putative advantages such as less pain after surgery, lesser hospital stay, and early return to normal activities compared to open surgery [2,3].

All the studies conducted in high-income countries have demonstrated the safety and effectiveness of laparoscopic surgery for rectal cancer. Some landmark trials including the COREAN trial revealed similar oncologic efficacy between laparoscopic and open rectal surgery while emphasizing reduced postoperative benefits with the laparoscopic surgery [4]. However, it is well recognized that results depend on considerations such as the surgeon’s experience, the volume of the institution, and compliance with oncological principles, which may differ between geographic areas and healthcare settings [5].

It has also been established that centers with a high volume provide optimal results from rectal cancer surgery because of the accumulated expertise and coordinated teamwork of the surgical staff [6]. Although these innovations have been reported, the application of laparoscopic techniques in the context of developing countries is still a major concern [7]. Different levels of surgeon experience, equipment accessibility, and patients’ selection criteria may become crucial for the result, which is why regional data can be needed to improve the organization of care [8,9].

Pakistan being a developing country has certain limitations in managing and offering high-quality surgical treatment of rectal cancer. There is scant information available in the literature concerning short-term results of laparoscopic rectal cancer operations at high-volume centers in the area. Peshawar itself is one of the prominent medical cities in the northern part of Pakistan and has multiple tertiary care centers and hence provides an opportunity to assess surgical performance in a low-resource environment. This study aims to offer important insights into the viability and efficacy of laparoscopic rectal cancer surgery in a developing country setting by examining factors such as postoperative complications, hospital stay, and early oncological outcomes.

## Materials and methods

This was a retrospective observational study carried out at the Surgical Oncology department, Shaukat Khanum Memorial Cancer Hospital and Research Center, Peshawar, a high-volume center for colorectal surgery. Assessment of short-term surgical results of laparoscopic rectal cancer operation ensued from April 1, 2021, to March 31, 2024. The sample comprised 209 patients with rectal cancer who were treated by laparoscopic rectal cancer surgery.

Inclusion criteria comprised of both genders of at least 18 years of age, with biomolecular confirmed rectal adenocarcinoma, curative intended patients with rectal cancer planned for laparoscopic surgery, tumor located within 15 cm of the anal verge either on preoperative imaging or endoscopic examinations, and patients with clinical staging of T1-T3, N0-N1, and M0 based on preoperative imaging (MRI or CT scan). Neoadjuvant chemoradiotherapy (NACRT) was indicated for patients with clinical stage T3, N0-N1, and M0 rectal adenocarcinoma. All patients received total neoadjuvant therapy, which included six cycles of neoadjuvant chemotherapy with capecitabine and oxaliplatin, followed by long-course chemoradiotherapy. A total radiation dose of 50 Gy was delivered in 25 fractions with concurrent capecitabine-based chemotherapy. The waiting period from the completion of NACRT to surgical intervention was approximately six to eight weeks to allow for maximum tumor downstaging. All patients completed the full course of NACRT. Exclusion criteria were the following: stage IV rectal cancer, that is, rectal cancer that has spread to other parts of the body, cancer patients who have had rectal cancer in the past, emergency surgical patients, patients with significant comorbidities, and those patients converted to open laparotomy before initiation of definitive dissection. The study was approved by the institutional review board of the hospital (ref. number EX-25-08-23-01 Annex-1).

All patients were managed according to enhanced recovery after surgery (ERAS) protocols, which included preoperative counseling, avoidance of prolonged fasting, carbohydrate loading up to two hours before surgery, and standardized multimodal pain management strategies (e.g., epidural analgesia or patient-controlled analgesia). Postoperative care included early ambulation, early enteral feeding, and thromboembolism prophylaxis.

Surgical technique

The laparoscopic rectal cancer surgeries followed a standardized approach to ensure oncological safety and optimal outcomes. Patients were put into the lithotomy position under general anesthesia following preoperative preparation, which included intestinal cleansing and antibiotic prophylaxis (Inj cefazolin 2 g and Inj metronidazole 500 mg). Pneumoperitoneum was established, and trocars were placed strategically to allow optimal access.

The operative steps began with an exploration of the abdomen in an effort to rule out metastatic disease, followed by a medial-to-lateral colonic mobilization of the sigmoid colon. Ligation of the inferior mesenteric artery and vein was performed for adequate lymphadenectomy, as well as splenic flexure mobilization if required for tension-free anastomosis. Total mesorectal excision (TME) was done using sharp dissection along the mesorectal fascia maintaining the hypogastric nerves and pelvic autonomic plexus. An endoscopic linear stapler was used to transect the rectum with margins based on tumor location. Stapled or handsewn anastomosis was performed and selective protective ileostomy was performed in patients with high risk for anastomotic complications. The specimen was extracted through a Pfannenstiel incision, and hemostasis was carefully secured.

ICU admission

Patients were admitted to the ICU based on predefined criteria, including hemodynamic instability, prolonged operative time (>4 hours), or the need for advanced postoperative monitoring. Postoperative complications were assessed using the Clavien-Dindo classification system. Cases with difficult parameters, such as obese patients, narrow pelvises, or radiation-induced fibrosis, received adjustments to optimize outcomes and keep homogeneity. All-cause mortality at 30 and 90 days after surgery and disease-free survival (DFS) were evaluated based on follow-up imaging and clinical evaluation.

Data were collected retrospectively from patient records. Recorded information included age, gender, comorbidities, tumor stage, tumor location, operative time, ICU stay, complications, relook procedures, mortality, and follow-up for DFS. Statistical analysis was performed with IBM SPSS Statistics for Windows, Version 25 (Released 2017; IBM Corp., Armonk, New York, United States). Descriptive statistics were used to summarize demographic and clinical data. Continuous variables were expressed as mean ± standard deviation (SD) or median (range), while categorical variables were reported as frequencies and percentages. Survival analysis was performed using the Kaplan-Meier method, and differences between groups were assessed using the log-rank test. Multivariate Cox regression analysis was used to control for confounding variables, including age, tumor stage, and microsatellite instability (MSI) status. Statistical significance was defined as a p-value < 0.05.

## Results

A total of 209 patients diagnosed with rectal cancer underwent laparoscopic rectal cancer surgery. The mean age of the patients was 43.07 ± 22.1 SD years, with an age range of 18-75 years. Patients were categorized into age groups: 18-39 years (30 patients, 14.3%), 40-59 years (106 patients, 50.7%), and 60-75 years (73 patients, 34.9%). The male-to-female ratio was 1.4:1, with males comprising 131 patients (62.7%) and females 78 patients (37.3%). The regional distribution included 54 (25.8%) patients from Afghanistan, 11 (5.2%) from the federally administered tribal area (FATA), 141 (67.5%) from Khyber Pakhtunkhwa (KPK), and three (1.4%) from Punjab. Preoperative assessment classified 191 (91.4%) patients as American Society of Anesthesiologists (ASA)-II and 18 (8.6%) cases as ASA-III. The most common tumor location was the mid rectum in 108 (51.7%) cases, followed by the upper rectum in 69 (33%) cases and the lower rectum in 32 (15.3%) cases. Preoperative carcinoembryonic antigen (CEA) levels were assessed in all patients, with elevated CEA levels (>5 ng/mL) observed in 45 patients (21.5%). Smoking status was also recorded, revealing that 62 patients (29.7%) were active smokers, 35 patients (16.7%) were former smokers, and 112 patients (53.6%) had never smoked. MSI status was evaluated in 180 patients (86.1%) with available tissue samples. Of these, 22 patients (12.2%) exhibited high microsatellite instability (MSI-H), while 158 patients (87.8%) were classified as microsatellite stable (MSS) or had low microsatellite instability (MSI-L) (Table 1).

**Table 1:** Demographics and other clinical data FATA: federally administered tribal area; KPK: Khyber Pakhtunkhwa; ASA: American Society of Anesthesiologists; CEA: carcinoembryonic antigen; MSI: microsatellite instability; MSI-H: high microsatellite instability; MSS: microsatellite stable; MSI-L: low microsatellite instability

Characteristics	Frequency	Percentage
Age groups		
18-39 years	30	14.3%
40-59 years	106	50.7%
60-75	73	34.9%
Mean age ± SD	43.07 ± 22.1	
Gender		
Male	131	62.7%
Female	78	37.3%
Regional distribution		
Afghanistan	54	25.8%
FATA	11	5.2%
KPK	141	67.5%
Punjab	3	1.4%
ASA classification		
ASA-II	191	91.4%
ASA-III	18	8.6%
Tumor location		
Mid rectum	108	51.7%
Upper rectum	69	33%
Lower rectum	32	15.3%
Preoperative CEA level		
>5 ng/ml	45	21.5%
Smoking status		
Active smoker	62	29.7%
Former smoker	35	16.7%
Never smoked	112	53.6%
MSI evaluation		
MSI-H	22	12.2%
MSS/MSI-L	158	87.8%

**Figure 1:**
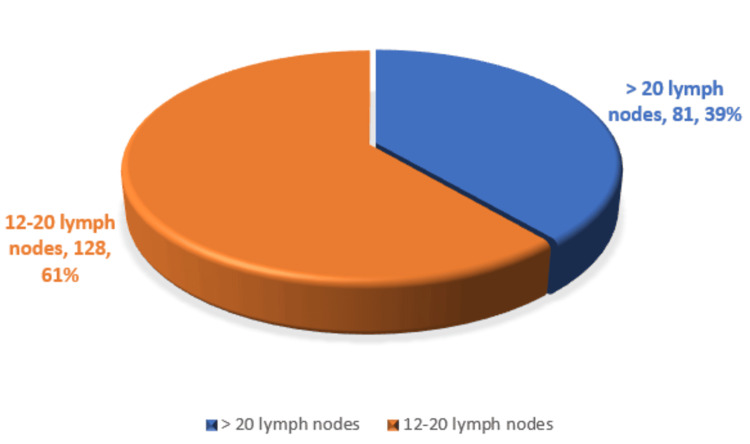
Lymph nodes reterieved

The number of lymph nodes retrieved during surgery ranged from 12 to 30, with 128 (61.2%) patients having 12-20 lymph nodes retrieved and 81 (38.7%) patients having > 20 lymph nodes retrieved (Figure 1).

**Figure 2:**
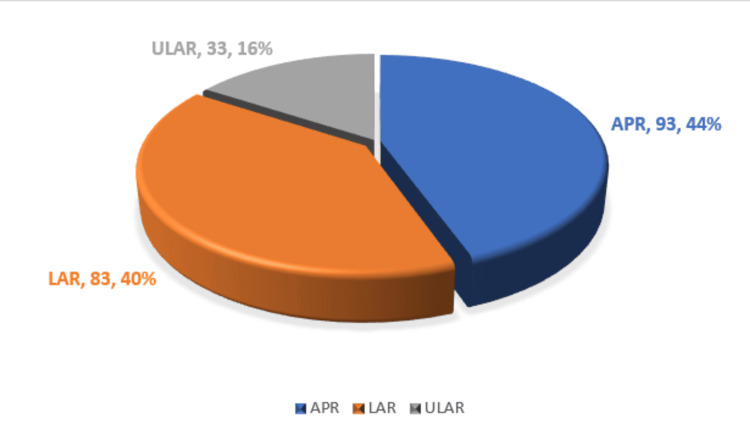
Surgical procedures performed

Abdominoperineal resection (APR) was the procedure of choice in 93 (44.5%) cases, followed by low anterior resection (LAR) in 83 (39.7%) cases and ultra-low anterior resection (ULAR) in 33 (15.8%) cases, respectively (Figure 2).

Histopathological type confirmed adenocarcinoma in 200 (95.7%) cases, mucinous adenocarcinoma in five (2.4%), adenosquamous carcinoma in two (1%), and leiomyosarcoma and malignant melanoma in one (0.5%) case each. Histopathological grading revealed well-differentiated tumors in 35 (16.7%) cases, moderately differentiated tumors in 161 (77%) cases, and poorly differentiated tumors in 13 (6.2%) cases, respectively. TNM staging showed stage I disease in 59 (28.2%) cases, stage II in 130 (62.2%) cases, and stage III in 20 (9.6%) cases, respectively (Table 2).

**Table 2:** Histopatholgy and TNM staging TNM: tumor, node, metastasis

Histological type	Number	Percentage
Adenocarcinoma	209	95.7%
Mucinous adenocarcinoma	4	2.4%
Adenosquameous carcinoma	2	1%
Leiomyosarcoma	1	0.5%
Malignant melanoma	1	0.5%
TNM stage-1	59	28.2%
TNM stage-II	130	62.2%
TNM stage-III	20	9.6%

The mean operative time was 190 ± 35 minutes, with only two cases (1%) requiring conversion to open surgery due to dense adhesions and locally advanced disease. Relook laparoscopy was necessary in three cases (1.4%) due to complications such as anastomotic leaks or sepsis. Postoperatively, 35 patients (16.7%) required ICU admission, with a median stay of two days (range: 1-5 days). The mortality rate was 0.5% (n = 1) at 30 days and remained the same at 90 days. DFS at 90 days, based on clinical follow-up and imaging, was observed in 170 patients (81.3%). Postoperative complications included surgical site infections (SSIs) in 13 patients (6.2%), anastomotic leaks in eight patients (3.8%), and reexploration in four cases (1.9%). Additionally, 10 patients (4.8%) required readmission due to various postoperative issues (Table 3).

**Table 3:** Intraoperative and postoperative outcome SSI: surgical site infection

Outcome	Value	Percentage
Operative time (mean±SD)	190 ± 35	
Conversion to open surgery	2	1%
Relook laparoscopy	3	1.4%
Need of ICU stay median: 2 days (range 1–5)	35	16.7%
Overall mortality	1	0.5%
Disease-free survival at 90 days	170	81.3%
SSI	13	6.2%
Anastomotic leak	8	3.8%
Reexploration	4	1.9%
Readmission	10	4.8%

## Discussion

Laparoscopic rectal cancer surgery has become the standard approach to rectal cancer treatment because of the benefits: less invasiveness, faster recovery, and similar oncological efficacy to the open method. This study adds to the expanding literature regarding laparoscopic rectal cancer approaches in well-selected patients and outlines the short-term experience of laparoscopic rectal cancer procedures in a high-volume tertiary care institution.

In our study, the mean operative time was 190 ± 35 minutes, similar to that reported in similar studies that found operative times between 180 and 220 minutes [10,11]. The conversion rate was 1%, which relates to the competence of the surgeons and the careful preoperative approach. Other high-volume centers have reported similar conversion rates as well (0.8%-3%), primarily due to locally advanced disease or dense adhesions [12].

Postoperative ICU stay was observed in 16.7% of cases, with a median duration of two days. This result aligns with findings from other studies that have utilized enhanced recovery protocols [13]. The 30-day and 90-day mortality rates of 1% and 2.4%, respectively, fall within the acceptable limits noted in the literature for laparoscopic rectal cancer surgeries, indicating that this technique can be safely implemented even in more complex situations [14].

In this study, the incidence of anastomotic leaks was found to be 3.8% (n = 8), while SSIs occurred at a rate of 6.2% (n = 13). Both figures are within the acceptable limits noted in existing literature [15]. These results underscore the importance of following standardized surgical protocols and enhanced recovery pathways. The anastomotic leak rate aligns with the reported range of 2%-6% and can be linked to the selective use of protective ileostomies in patients deemed at high risk [16]. Additionally, the application of advanced surgical techniques and a commitment to oncological principles likely played a significant role in achieving these positive outcomes.

In laparoscopic surgeries, oncologic safety is primarily gauged through lymph node yield, negative circumferential resection margins, and TME quality. This study indicates that 38.7% of patients had a lymph node yield > 20, satisfying the recommended oncological standard. The distribution of TNM stages (stage I, 28.2%; stage II, 62.2%; stage III, 9.6%) was indicative of efficient preoperative staging and selection. Comparable lymph node retrieval and staging distribution have been reported by Stevenson et al. and Dobbins et al. [17,18].

The 90-day DFS rate was 81.3%, which is encouraging and indicates adequate cancer eradication. Although long-term results are not yet available, short-term DFS rates are consistent with findings from pivotal trials such as COLOR II, which reported 85% DFS at six months [19].

The surgical technique used in this study showed the superiority of abdominoperineal resection. The choice of procedure was based on tumor location, patient factors, and expertise of the surgeon. Notably, LAR and ULAR were performed in 39.7% and 15.8% of cases, respectively, mainly for distant tumors requiring extended margin removal. This is in line with the reports of Fleshman et al. who emphasized its role in achieving negative gross margins [20].

Strengths

The study is conducted in a high-volume center, improving the reliability of its findings. A relatively large sample size (n = 209) enhances statistical power. The use of a standardized surgical technique improves the validity of comparisons. Reporting important oncological outcomes such as lymph node retrieval and DFS provides meaningful insights.

Limitations

The single-center design limits the generalizability of our findings. The short-term follow-up period of 90 days does not provide an understanding of long-term oncological outcomes. The absence of a control group restricts comparative effectiveness analysis. Additionally, while all patients received long-course NACRT, we did not comprehensively report the clinical response rates to NACRT. Decisions regarding further management for complete versus nonresponders were not fully explored. Lastly, the absence of a complication management strategy reduces the clinical applicability of our results. To overcome these limitations, further future randomized controlled trials (RCTs) and prospective clinical research with longer follow-up periods and multiple, multicenter cohorts are warranted.

## Conclusions

This study shows that laparoscopic rectal cancer surgery is a safe and effective approach with low conversion rates, minimal morbidity, and favorable short-term oncological outcomes in a high-volume center. Adequate lymph node retrieval and tumor-free margins confirm its oncological safety, while the low rates of postoperative complications highlight its procedural reliability. Further multicenter studies with long-term follow-up are essential to establish its role as the standard of care for rectal cancer.

## References

[1] Sung H, Ferlay J, Siegel RL, Laversanne M, Soerjomataram I, Jemal A, Bray F (2021). Global cancer statistics 2020: GLOBOCAN estimates of incidence and mortality worldwide for 36 cancers in 185 countries. CA Cancer J Clin.

[2] Shah MF, Nasir IU, Ahmad R, Ahmad S, Amjad A, Zaineb KB, Rehman R (2024). Short-term outcomes of first 100 laparoscopic colorectal surgeries at a newly developed surgical setup at Peshawar. Cureus.

[3] (2009). Screening methods for early detection of colorectal cancers and polyps: summary of evidence-based analyses. Ont Health Technol Assess Ser.

[4] Kang SB, Park JW, Jeong SY (2010). Open versus laparoscopic surgery for mid or low rectal cancer after neoadjuvant chemoradiotherapy (COREAN trial): short‐term outcomes of an open‐label randomised controlled trial. Lancet Oncol.

[5] Lacy AM, Delgado S, Castells A, Prins HA, Arroyo V, Ibarzabal A, Pique JM (2008). The long-term results of a randomized clinical trial of laparoscopy-assisted versus open surgery for colon cancer. Ann Surg.

[6] Katella K (2023). Colorectal cancer: what millennials and gen zers need to know. Yale Medicine.

[7] Murphy CC, Singal AG, Baron JA, Sandler RS (2018). Decrease in incidence of young-onset colorectal cancer before recent increase. Gastroenterology.

[8] Deijen CL, Vasmel JE, de Lange-de Klerk ES (2017). Ten-year outcomes of a randomised trial of laparoscopic versus open surgery for colon cancer. Surg Endosc.

[9] Guillou PJ, Quirke P, Thorpe H, Walker J, Jayne DG, Smith AM (2005). Short‐term endpoints of conventional versus laparoscopic‐assisted surgery in patients with colorectal cancer (MRC CLASICC trial): multicentre, randomised controlled trial. Lancet.

[10] Done JZ, Fang SH (2021). Young-onset colorectal cancer: a review. World J Gastrointest Oncol.

[11] Luglio G, Nelson H (2010). Laparoscopy for colon cancer: state of the art. Surg Oncol Clin N Am.

[12] Kim SJ, Ryu GO, Choi BJ, Kim JG, Lee KJ, Lee SC, Oh ST (2011). The short-term outcomes of conventional and single-port laparoscopic surgery for colorectal cancer. Ann Surg.

[13] Ahmad R, Abbasi HJ, Nasir IU, Shah MF (2024). Demographic characteristics and short-term outcomes of laparoscopic colon cancer surgeries at a newly developed cancer center in Peshawar, Pakistan. Pak J Med Sci.

[14] Chan AC, Law WL (2007). Outcome of laparoscopic surgery in colorectal cancer: a critical appraisal. Expert Rev Pharmacoecon Outcomes Res.

[15] Phillips EH, Franklin M, Carroll BJ, Fallas MJ, Ramos R, Rosenthal D (1992). Laparoscopic colectomy. Ann Surg.

[16] Braga M, Frasson M, Vignali A, Zuliani W, Capretti G, Di Carlo V (2007). Laparoscopic resection in rectal cancer patients: outcome and cost-benefit analysis. Dis Colon Rectum.

[17] Stevenson AR, Solomon MJ, Brown CS (2019). Disease‐free survival and local recurrence after laparoscopic‐assisted resection or open resection for rectal cancer: the Australasian laparoscopic cancer of the rectum randomized clinical trial. Ann Surg.

[18] Dobbins TA, Young JM, Solomon MJ (2014). Uptake and outcomes of laparoscopically assisted resection for colon and rectal cancer in Australia: a population-based study. Dis Colon Rectum.

[19] Buunen M, Bonjer HJ, Hop WC (2009). COLOR II. A randomized clinical trial comparing laparoscopic and open surgery for rectal cancer. Dan Med Bull.

[20] Fleshman J, Sargent DJ, Green E (2007). Laparoscopic colectomy for cancer is not inferior to open surgery based on 5-year data from the COST study group trial. Ann Surg.

